# 

**Published:** 1918-04-20

**Authors:** 


### CONTENTS.

PAGE

**The New Man-Power Scheme** ... ... ... 41

**New President of Royal College of Physicians** 42

**H.M. King George at Lincoln** ... ... ... 43

**Hospital and Institutional News** ... ... ... 43

Not Killed but a Prisoner—The War Office and Bristol Royal Infirmary—A New Hospital for London—Chichester and Sectional Subscriptions—An Opportunity for Aberdeen—The Case for a Higher Government Grant—A Hospital Estate—Three Gifts to the Ramsgate General Hospital—Proposed Stanley Maude Convalescent Home—Pay Wards in Cottage Hospitals—The Red Cross Sale and Queen Mary's Exhibition—Gifts of Plate from Grateful Patients—Words and Things: A Case in Point—Exposure During Air-raids—An Air-raid Cot—St. John Ambulance Brigade at Birmingham—Consumptives and the Food Rations—Patients and Sanatoriums—Municipal Work to Ensure Pure Milk—The Status of Dental Surgery—A Healthy War Hospital—Modern Books for Soldiers—Diphtheria Cases in Devonshire—The Salaries of Masseuses—Two April *Gazettes*—The British Pharmacopœia in War-time—This Week's Drug Market.

**The Great War** ... ... ... ... ... ... 49

VI. The Progress of the Sick.

**Medicine and Politics** ... ... ... ... ... 51

**More Scottish Asylum Reports** ... ... ... 52

Germans and Others Behind the Age.

**Hospital Life in Macedonia** ... ... ... ... 54

PAGE

**Nature Notes from Palestine** ... ... ... 53

The Valley of Ajalon.

**Essex County Hospital** ... ... ... ... 55

Important Letter from the Chairman, with the Report of the Medical Board.

**The Editor's Letter-Box** ... ... ... ... 57

"What Nurses should Seek and Find"—"Hahnemann and Public Confidence."

**An Imperial Union of Nurses** ... ... ... 58

Pernicious Propaganda.

**The Matrons' and Sisters' Department** ... 59

The Growth of the Nursing Profession: VII. The Employment of the Waiting Years.

Round the Hospitals.

Queen Alexandra at St. Paul's—Notable Facts of College Progress—Matrons on Municipal Committees—Important Gift to R.N.P. Fund—L.C.C. Lectures on Domestic Science—Nurses' Homes without Table-cloths.

**The College of Nursing (Limited by Guarantee)** 63

List No. 6 of Training Schools of Registered Nurses.

**Public Health Nursing** ... ... ... ... 63

A Volume to be Read and Studied.

**Matrons' and Sisters' Appointments** ... ... 64

**Some Annual Meetings** ... ... ... ... 65

**Hospital Finance in 1917** ... ... ... ... 65

**The Institutional Worker** ... ... (Suppl.) 3

